# The complete chloroplast genome sequence of *Callitriche palustris* (Plantaginaceae)

**DOI:** 10.1080/23802359.2021.1967802

**Published:** 2021-08-30

**Authors:** Jiaojun Yu, Hongjin Dong

**Affiliations:** a Hubei Key Laboratory of Economic Forest Germplasm Improvement and Resources Comprehensive Utilization, Huanggang Normal University, Huanggang, China; b Hubei Collaborative Innovation Center for the Characteristic Resources Exploitation of Dabie Mountains, Huanggang, China

**Keywords:** *Callitriche palustris*, complete chloroplast genome, Plantaginaceae phylogeny

## Abstract

*Callitriche palustris* L. is an annual aquatic or marsh plant, wide spread in temperate regions throughout the world. In present study, we sequenced, assembled and annotated the complete chloroplast (cp) genome of *C. palustris*. The length of *C. palustris* complete cp genome was 150,138 bp, with a typical quadripartite structure comprising a pair of inverted repeat regions (IRs; 25,667 bp), a large single copy region (LSC; 81,432 bp) and a small single copy region (SSC; 17,372 bp). The whole cp genome contained 134 genes, including 89 protein-coding genes (PCGs), 37 transfer RNA (tRNA) genes, and eight ribosomal RNA (rRNA) genes. The maximum likelihood (ML) phylogenetic analysis indicated that *C. palustris* was a member of Plantaginaceae, but the relationships between subfamilies and tribes need more samplings. This cp genome would provide a valuable genetic resource for *C. palustris*’ phylogenetic study.

*Callitriche palustris* L. is an annual aquatic or marsh plant, with very small flowers in the axillary. It is wide spread in temperate regions throughout the world, in the altitude from sea level to 5000 m (Min and Lansdown 2008). Pollination of the aquatic *Callitriche* may be by wind (above the lake surface) or by water, or there may be self-pollination (Martinsson 1993; Osborn et al. 2001). *Callitriche* was the only representative in Callitrichaceae, but now is placed in the family Plantaginaceae based on three plastid genes (*rbcL*, *ndhF*, and *rps2*) (Osborn et al. 2001).

The voucher specimen of *C. palustris* used in this study were collected from Bashui, Huangzhou, Hubei, China (115°22′03.31″E, 31°11′19.92″N, 20 m, in the pool). The collected specimen was deposited in Herbarium of Huanggang Normal University (HGTC, former Herbarium of Biology Department of Huanggang Teachers College, Mr. Jun Fu, fujun@hgnu.edu.cn) under voucher number 2018-12-1 (collected by *Dong Hongjin* et al.). Genomic DNA was extracted from fresh leaves of a seedling according to a modified CTAB method (Doyle and Doyle 1987). Total genome DNA of *C. palustris* was sequenced by Illumina Hiseq 2500 Sequencing System (Illumina, Hayward, CA) with 150 bp paired-end. The reads were assembled through the GetOrganelle software (Jin et al. 2020). The complete cp genome of *C. palustris* was annotated with software PGA (Qu et al. 2019) and Geneious ver. 10.1 (http://www.geneious.com (Matthew et al. 2012) and then submitted to GenBank (accession no. MW774642). The genome annotation was performed by aligning with the cp genomes of relatively related species.

The size of cp genome of *C. palustris* is 150,138 bp, including a large single-copy (LSC) region of 81,432 bp and a small single-copy (SSC) region of 17,372 bp separated by a pair identical inverted repeat regions (IRs) of 25,667 bp each. A total of 134 genes were successfully annotated containing 89 protein-coding genes, 37 tRNA genes, and eight rRNA genes. GC content of the whole genome, IRs, LSC and SSC regions are 37.8%, 43.1%, 37.8%, and 31.0%, respectively. GC content of IRs region is the highest. Twenty-one genes contain one intron, while two genes have two introns. The complete cp genome sequence of *C. palustris* and other species from Plantaginaceae and close relatives were used to construct phylogenetic tree (Figure 1). The sequences were initially aligned using MAFFT (Kazutaka and Standley 2013) and then visualized and manually adjusted using BioEdit (Hall 1999). Take the plastome of *Saxifraga stolonifera* (GenBank: MN496079) as an out-group, a maximum likelihood analysis was performed with RAxML version 8 program (Alexandros 2014) using 1000 bootstrap. IQ-tree was also used to construct ML tree with fast mode (Nguyen et al. 2015). The result supports that *C. palustris* was a member of Plantaginaceae, consistent with the previous studies (Philbrick and Les 2000; Olmstead et al. 2001). The relationships between subfamilies and tribes need more samplings.

**Figure 1.**
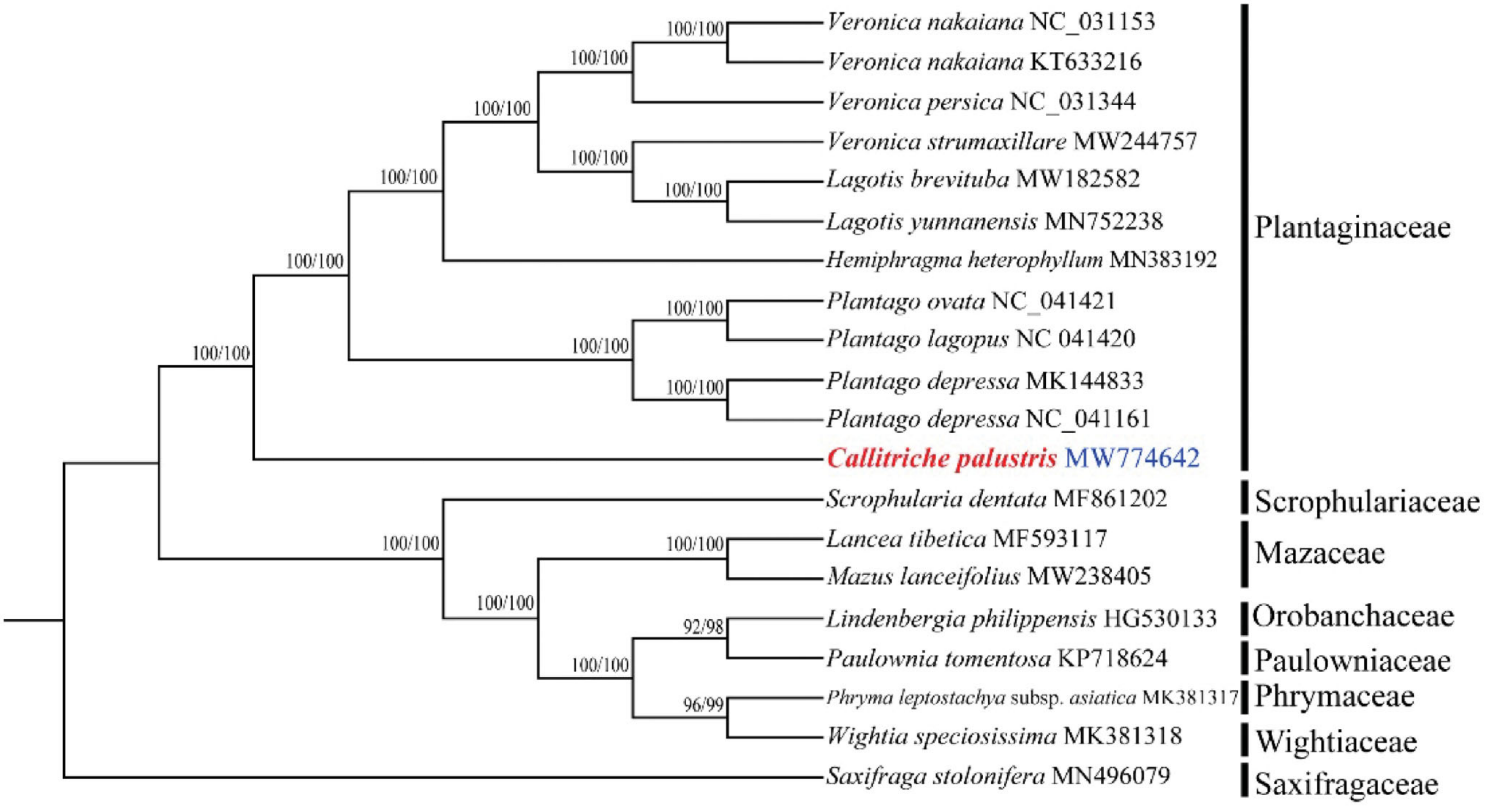
Maximum likelihood phylogenetic tree for *Callitriche palustris* based on complete cp genomes. The number on each node indicates bootstrap support value generated by RaxML/IQ-tree.

## Data Availability

The data that support the findings of this study are available in GenBank of NCBI at https://www.ncbi.nlm.nih.gov, accession number MW774642. The assembled individual was linked with no. SAMN18324991 and Project ID: PRJNA715046. Raw sequencing reads used in this study were deposited in the GenBank database of Sequence Read Archive with no. SRR14844933.
